# The Role of Magnetic Resonance Pulse Sequences in the Diagnosis of Acute Appendicitis in Pregnant Women

**DOI:** 10.7759/cureus.51312

**Published:** 2023-12-30

**Authors:** Thien Thanh Thi Nguyen, Huyen Mai Duy Le, Duy Thanh Nguyen, Hieu Quang Nguyen, Nam Hoang Nguyen, Duc Tan Vo, Chien Cong Phan

**Affiliations:** 1 Department of Diagnostic Imaging, University Medical Center of Ho Chi Minh City, Ho Chi Minh, VNM

**Keywords:** diagnosis, appendicitis, pregnancy, sequence, magnetic resonance imaging

## Abstract

Introduction: Acute appendicitis (AA) is one of the most common surgical emergencies, with a lifetime risk estimated at 7-8%. Pregnant women with appendicitis can have a difficult diagnosis because many signs and symptoms could overlap with other causes of acute abdominal pain. Although magnetic resonance imaging (MRI) is not contraindicated at all gestational ages for units with a field strength of three Tesla or less, there is still much discussion regarding the best protocol to follow in order to minimize survey time and maximize diagnostic efficiency. The purpose of this study was to assess how well different MR pulse sequences can diagnose AA.

Methods: This retrospective study involved 179 pregnant females. All patients treated and admitted to the University Medical Center, Ho Chi Minh City, Vietnam, between January 2016 and October 2023 had their MR scans and medical data examined. MRI results were assessed and compared with surgical and histopathological findings.

Results: The mean age of the population was 29.7 ± 4.8 years (range, 18-46 years). On T1-weighted (T1W) and T2-weighted (T2W) sequences, the appendix was clearly visualized at rates of 81.8% and 89.9%, respectively. The sensitivity and specificity of the T2W in diagnosing AA were 93.5% and 92.3%, and when combined with T1W and diffusion-weighted (DW) images, the sensitivity and specificity further increased, being 96.8% and 94.9%, respectively. The predictive value of non-AA of the T1 bright appendix sign was 95.6%.

Conclusion: Our study supports the use of MRI as an imaging test to identify appendicitis during pregnancy, as it has been shown to be a useful method for diagnosing the condition in pregnant women. The T2W pulse sequence is a useful tool for diagnosing appendicitis because of its high sensitivity and specificity. When identifying appendicitis from T2W alone proves challenging, T1W with the T1 bright sign and DW to take advantage of the appendix lumen and/or wall's diffusion features can yield additional information and boost diagnostic confidence.

## Introduction

Acute appendicitis (AA) is one of the most common surgical emergencies, with a lifetime risk estimated at 7-8% and a reported incidence of one in every 15 individuals [1,2]. Clinical manifestations of AA in gravid women are diverse and easily confused with other diseases because of overlapping symptoms and signs, making diagnosis challenging. Moreover, diagnosing the cause of acute abdominal pain in pregnant women is a challenge due to many factors, such as nonspecific leukocytosis, physiologic changes, obstetric problems, and organ location changes due to uterine enlargement. Ultrasound is the first tool in diagnosing AA; however, the field of investigation is limited when the uterus is enlarged, making it challenging to locate the appendix. Although computed tomography is a highly accurate method of diagnosing appendicitis with a sensitivity and specificity above 90%, according to some studies [3-5], the diagnosis of appendicitis in pregnant women is made more challenging due to radiation exposure as a concern.

Magnetic resonance imaging (MRI) plays a vital role in diagnosing appendicitis in pregnant women because of its multiplanar capability and devoid of ionizing radiation. The World Society of Emergency Surgery 2020 recommendation stated that, if an MRI was available, it should be used in pregnant patients with suspected appendicitis following an inconclusive ultrasound [6]. The sensitivity and specificity of MRI in diagnosing AA have been reported at 90-100% and 94-98%, respectively [7-10]. Although there is currently no evidence that magnetic fields have specific effects on the fetus and, according to clinical guidelines, MRI is not contraindicated at all gestational ages for units with a field strength of three Tesla or less, establishing the most effective protocol to help reduce specific absorption rates and achieve the highest diagnostic efficiency is essential and still not debated enough [11,12]. That is why we conducted the current research on the role of MR pulse sequences in diagnosing AA.

## Materials and methods

Study design

This is a cross-sectional, retrospective study involving 179 pregnant females.

Data collection

Subjects

The medical records and MR images of all patients admitted and treated at University Medical Center of Ho Chi Minh City, Vietnam, from January 2016 to October 2023 were retrospectively reviewed. Data regarding demographics and pathologic findings were collected and analyzed.

Inclusion and Exclusion Criteria

All pregnant patients (determined by imaging or beta-human chorionic gonadotropin test) over 18 years of age suspected of AA and having an MRI with the appendix protocol were included in this study. The gestational age was 22.6 ± 8 weeks (range, 6-39 weeks). Pathological evidence was available if the patient had an appendectomy. In cases without surgery, the patient was followed until AA was ruled out. We excluded cases when the image quality was not guaranteed, patients with claustrophobia, those who had implants that were unsafe, or when patients could not be monitored for the final diagnosis.

Based on clinical data and surgical and pathological reports, the patients were divided into AA and non-AA groups. The AA group included patients whose surgical and pathological reports both confirmed the presence of AA. The non-AA group included patients who underwent surgery to confirm a normal appendix or had follow-ups to rule out AA if there were no surgeries carried out.

MR Protocol

The MR studies were performed on either a 1.5 T (Magnetom Avanto, Siemens Healthcare Limited, Germany) or 3.0 T (Magnetom, Siemens Healthcare Limited, Germany) MR scanner using a phased-array surface coil with six channels covering the abdomen and pelvis without using an oral contrast material.

A comprehensive multiplanar protocol was used for evaluating the appendix. The sequences obtained were as follows: axial, sagittal, and coronal T2-weighted (T2W) half-Fourier single-shot turbo spin-echo (HASTE); axial T2W HASTE short tau inversion recovery (TIRM); axial T1-weighted (T1W) volume interpolated breath-hold (VIBE) DIXON without gadolinium; and diffusion-weighted (DW) sequence. The parameters of the pulse sequences used are shown in Table 1.

**Table 1:** Parameters of the pulse sequences TR: repetition time, TE: echo time, FOV: field of view, TA: acquisition time, T2W: T2-weighted, T1W: T1-weighted, HASTE: half-Fourier single-shot turbo spin-echo, TIRM: short tau inversion recovery, VIBE: volume interpolated breath-hold, DW: diffusion-weighted, b: b-value of the diffusion-weighted sequence

Sequence	TR (ms)	TE (ms)	FOV (cm)	Matrix	Slice thickness (mm)	Gap (mm)	TA (second)
T2W HASTE coronal	1300	100	35	256x205	4	0.8	38
T2W HASTE axial	1300	100	38	320x256	4	0.8	39
T2W HASTE sagittal	1300	100	35	256x205	4	0.8	38
T2W HASTE TIRM axial	1000	116	36	256x230	4	0.8	36
T1W VIBE DIXON	4.16	1.34	38	320x240	3	0.6	10
DW (b = 50, b = 800)	2200	70	32	124x96	3	0.3	44

Image Analysis

Within the Picture Archiving and Communication System (PACS), two radiologists with over five years of expertise in abdominopelvic imaging independently analyzed and reported MR images. In cases where there were differences of opinion between the two radiologists, the assessment of a senior radiologist was considered the conclusion.

**Figure 1:**
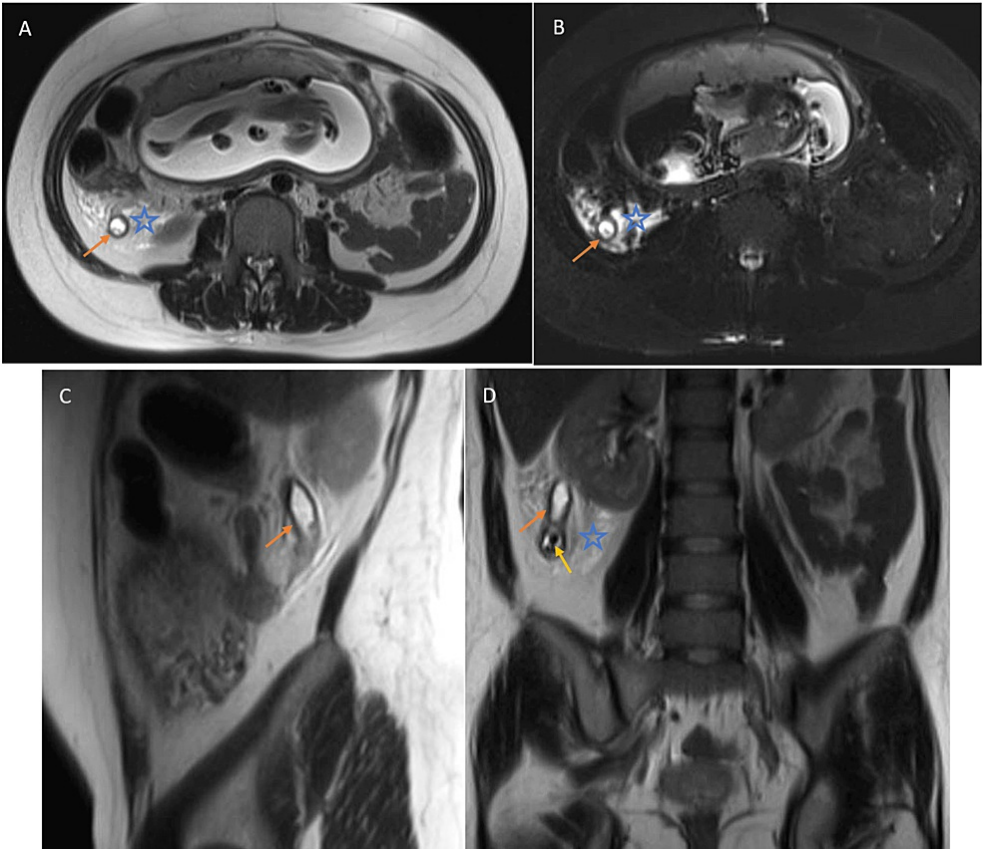
Appendicitis on T2W sequences T2-weighted (T2W) (A, C, D) and T2W half-Fourier single-shot turbo spin-echo (HASTE) short tau inversion recovery (TIRM) (B) images of a proven case of acute appendicitis in a 32-year-old woman at a 20-week gestation with acute epigastric pain migrating to the right iliac fossa. The dilated and fluid-filled inflamed appendix was well visualized (orange arrow). Appendicolith (yellow arrow) and peri-appendiceal stranding (star) were also apparent.

**Figure 2:**
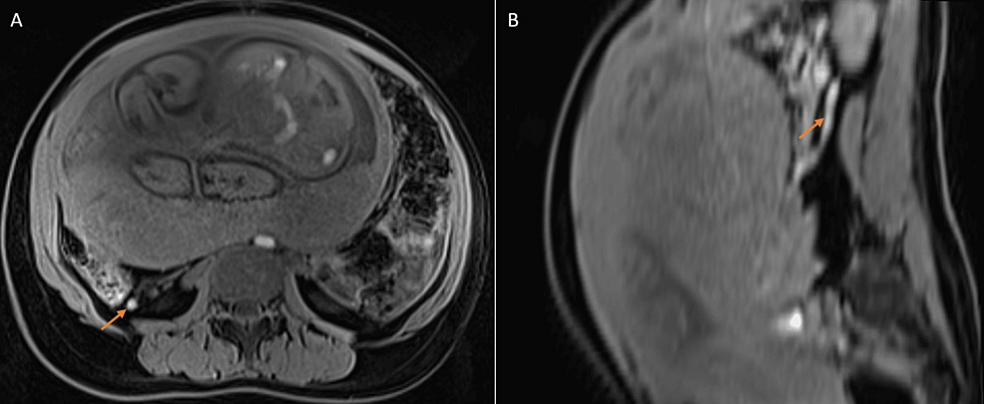
T1 bright appendix sign Axial (A) and reconstructed sagittal (B) T1W images showing the positive T1 bright appendix sign (arrow) in a case later confirmed as no appendicitis through follow-up in a 28-year-old female at 33-week-four-day gestation with acute right lower quadrant pain.

The location and diameter of the appendix were assessed for each patient. We classified the patients into three groups based on MR findings: (1) “definite appendicitis” if the diameter was larger than 10 mm or the diameter was from 6 to 10 mm and the appendix had all of the accompanying signs of inflammation (Figure 1); (2) “suspected appendicitis” if the diameter of the appendix was from 6 to 10 mm; (3) “normal or non-visualized appendix.” Besides the appendiceal diameter, the accompanying MR signs of inflammation included appendiceal wall thickening ≥2 mm, high signal luminal contents on T2W images (due to fluid), appendicolith, peri-appendiceal fat stranding or edema, peri-appendiceal or right lower quadrant fluid, and appendiceal restricted diffusion. The peri-appendiceal edema was best visualized on the T2 HASTE TIRM sequences. Appendiceal restricted diffusion was regarded as having a high signal on DWI (high b value images) and a correspondingly low signal ​​on the apparent diffusion coefficient (ADC) map. In addition, the T1 appendix bright sign was defined as an area of linear high T1 signal intensity filling more than half the length of the appendix.

Statistical analysis

Data are shown as mean values ± standard deviation for normal distribution or median and interquartile range if data are not normally distributed. Qualitative variables are described as numbers and percentages. A 2×2 contingency table was used to determine the sensitivity, specificity, positive predictive value (PPV), and negative predictive value (NPV) for every MRI characteristic. Stata Statistical Software, release 17 (2021, StataCorp LLC, College Station, TX) was used for all analyses.

Ethical considerations

This study was conducted at the University Medical Center of Ho Chi Minh City, in accordance with the Declaration of Helsinki. The protocol was approved by the Human Research Ethics Committee of the University Medical Center of Ho Chi Minh City. The written informed consent was waived by the Human Research Ethics Committee of the University Medical Center of Ho Chi Minh City. The approval number is 118/GCN-HDDD, dated December 5, 2021.

## Results

Participant characteristics

The current study included 179 pregnant patients. The mean age of the population was 29.7 ± 4.8 years (range, 18-46 years), and the gestational age of the fetus was 22.6 ± 8 weeks (range, 6-39 weeks).

Imaging features

The T2W images were examined in all cases, the T1W images in 170 (95%), and the DW pulse sequences in 65 (36.3%) of the patients (Table 2).

**Table 2:** Distribution of pulse sequences in the acquisition protocol T2W: T2-weighted, T1W: T1-weighted, DWI: diffusion-weighted imaging

	Number	Percentage (%)
T2W	179	100
T1W	170	95.0
DWI	65	36.3

The rates of clear visualization of the appendix on T1W and T2W were 81.8% and 89.9%, respectively. Compared to using T2W images alone, combining T1W and T2W images can help visualize six more cases (3.4%) (Table 3).

**Table 3:** Rate of appendix visualization on each pulse sequence n: number of cases, T1W: T1-weighted, T2W: T2-weighted

	T1W (n, %)	T2W (n, %)	Combined
Clear	139 (81.8)	161 (89.9)	167 (93.3)
Unclear	23 (13.5)	13 (7.3)	7 (3.9)
Not detected	8 (4.7)	5 (2.8)	5 (2.8)
Total	170	179	179

The role of pulse sequences in diagnosing AA is demonstrated in Table 4. The sensitivity and specificity of the T2W were 93.5% and 92.3%, respectively, and when combined with the T1W and DW images, the sensitivity and specificity further increased, being 96.8% and 94.9%, respectively. T1W and DW pulses helped increase diagnostic capabilities.

**Table 4:** Role of pulse sequences in the diagnosis of acute appendicitis n: number of cases, T1W: T1-weighted, T2W: T2-weighted, DW: diffusion-weighted

	T2W	T2W combined with T1W/DW
AA group (n, %)		
Clear	58 (93.5)	60 (96.8)
Suspected	4 (6.5)	2 (3.2)
Normal	0 (0)	0 (0)
Total	62	62
Non-AA group (n, %)		
Clear	0 (0)	0 (0)
Suspected	9 (7.7)	6(5.1)
Normal	108 (92.3)	111 (94.9)
Total	117	117

The T1 bright appendix sign helped exclude appendicitis but had low sensitivity (38.1%, 43/113). On the other hand, the AA group had two cases with the T1 bright appendix sign, accounting for 3.5% (2/57) (Figure 2).

The positive predictive value (prediction of non-AA) of the T1 bright appendix sign was 95.6% (43/45) (Table 5).

**Table 5:** Distribution of the T1 bright appendix sign in the two groups n: number of cases

	AA group (n, %)	Non-AA group (n, %)
Bright	2 (3.5)	43 (38.1)
No	55 (96.5)	70 (61.9)
Total	57	113

**Figure 3:**
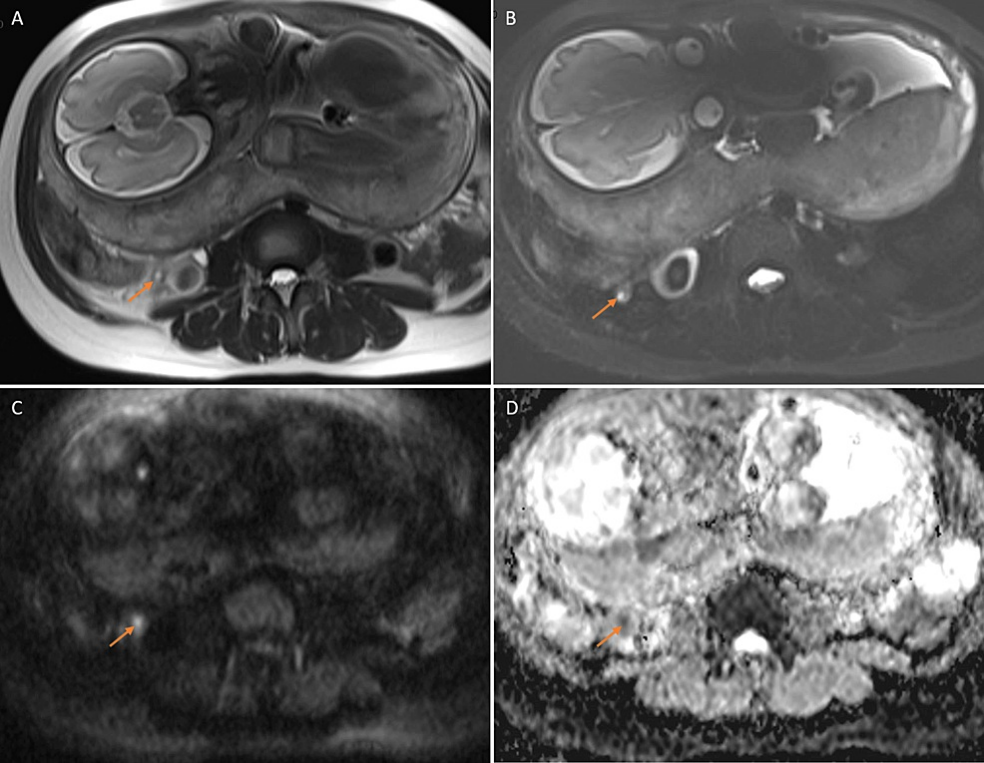
Appendicitis on T2W and DW sequences T2W (A), T2W HASTE TIRM (B), high b-value (800 s/mm2) DW (C), and ADC map (D) images showing the supporting value of diffusion images in characterizing the appendix in a case of surgically and pathologically confirmed appendicitis with undetermined features on T2W images. The appendix (arrow) had a diameter of 8 mm and very mild surrounding stranding. Appendiceal restricted diffusion was evident: hyperintense on DW images and correspondingly hypointense on the ADC map. T2W: T2-weighted, HASTE TIRM: half-Fourier single-shot turbo spin-echo short tau inversion recovery, DW: diffusion-weighted, ADC: apparent diffusion coefficient

In the AA group, 20 of the 27 cases that integrated the DWI into the protocol demonstrated restricted diffusion of the appendix (Figure 3).

## Discussion

AA in pregnant women is a challenge in which MRI is an increasingly proven tool to assess its presence, especially in cases where ultrasound was inconclusive.

Our study showed that MRI had a high sensitivity and specificity for identifying AA in pregnant women; when T1W, T2W, and DWI sequences are combined, these values increased to 96.8% and 94.9%, respectively. According to some studies, MRI was reported to have a sensitivity of 91.8-100% and a specificity of 97.9-99.9% [9,13-15]. Demonstrating similar results to other research, we propose that in pregnant patients suspected of AA, MRI can be utilized as an imaging modality of choice, especially when the ultrasound diagnosis is unclear [9,16].

While MRIs at all gestational ages with field strengths of three Tesla or less are not contraindicated, it is crucial to design an ideal protocol that will allow for early disease diagnosis and shorten acquisition times while still achieving MRI safety standards like the lowest specific absorption rate. Our study showed that the T2W is a handy pulse sequence with high sensitivity and specificity (93.5% and 92.3%, respectively) to diagnose AA in gravid females. When combining T2W images with T1W and/or DW images, the sensitivity and specificity increased (96.8% and 94.9%, respectively).

According to our study, T1W images can help increase the visibility of the appendix. This may be due to its inherent thin slice thickness, which was helpful when the patient’s mesenteric fat was abundant. The T1 bright sign was specific for the diagnosis of non-AA appendix in suspected pregnant patients; however, it was seen in only 38.1% (43/113) non-AA patients. Classically, a normal appendix could be identified when the diameter was less than 6-7 mm, the intraluminal was T2 hypointense, and a blooming artifact was visible on T2*-weighted images due to intraluminal air. The T1 bright signal might have originated from the short T1 relaxation times of stools and concentrated and dehydrated food materials. In cases of uncertain appendicitis on T2W and T1W images, the DW pulse sequence helped increase the confidence in diagnosing AA in two more cases by exploiting the diffusion features of the appendix lumen and/or wall. Diffusion imaging depicts the motion of water protons, needs no contrast material administration, and has a relatively short scan time. A study reported that the thick appendiceal wall and increased signal in peri-cecal fat were more noticeable on DWI. Diffusion restriction has been reported in inflammatory processes, as in the case of AA. The addition of these relatively rapidly acquired pulse sequences improves MRI performance for the diagnosis of AA during pregnancy.

**Figure 4:**
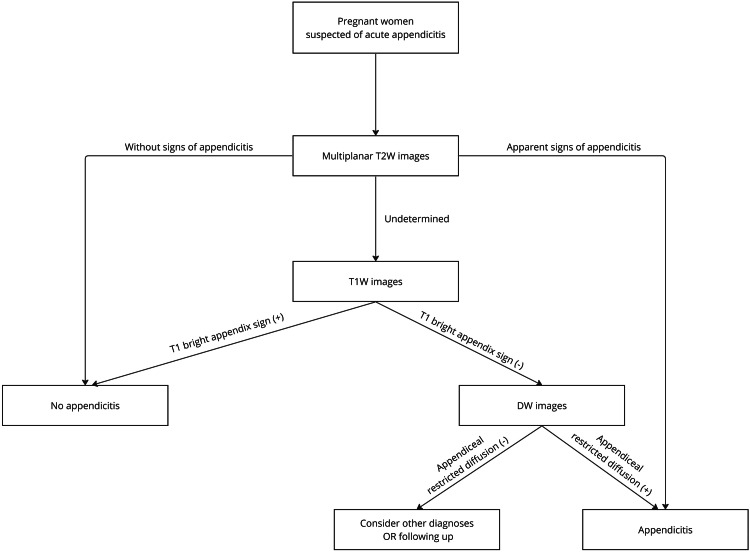
Flow chart for the MR pulse selection Our suggested MR pulse sequence selection algorithms optimize the scan time. Multiplanar T2W images were performed on pregnant patients suspected of AA. The scan will be stopped when AA can be determined with confidence from the T2W images. If the T2W images are inconclusive, T1W pulse sequences will be performed. The scan will be stopped if there is no evidence of inflammation and the bright T1W appendix sign is present. DW will be used to aid in the diagnosis if the obtained T2W and T1W images are still unclear.

The goal of this study is to optimize the protocol to limit the scan duration while still being sufficient for diagnosis. We suggest an algorithm to perform an MR scan on suspected AA individuals. When AA can be confidently concluded from the T2W images, the scan can be stopped. If the T2W images are inconclusive, T1W pulse sequences will be performed. If the bright T1W appendix sign is present and signs of inflammation are absent, the scan will be stopped. If the acquired T2W and T1W images are still uncertain, DW will be performed to assist the diagnosis in some cases and characterize the diffusion property of fluid within the appendix lumen and the appendiceal wall (Figure 4).

Specific limitations should be acknowledged. First, being a retrospective observational analysis, selection bias was present. Second, patients with proven AA on ultrasound may not be included in the study population.

## Conclusions

Diagnosing appendicitis in pregnant women is challenging due to the numerous overlapping signs and symptoms. Therefore, a safe and accurate imaging modality is crucial to patient management strategies. MRI has been proven to be a valuable technique in diagnosing appendicitis in pregnant women, and our study supports the utilization of MRI as an imaging test to diagnose appendicitis during pregnancy when ultrasound is inconclusive or a clinical diagnostic dilemma exists. Moreover, retrieving MRI with various pulse sequences can occasionally be challenging for radiologists, which adds time to the image interpretation process. As a result, it is required to suggest a flow chart for a strategy approach.

In MR pulse sequences, T2W is a handy pulse sequence with high sensitivity and specificity for diagnosing appendicitis. In cases where T2W alone images are difficult to determine, T1W with the T1 bright sign and DW exploiting the diffusion features of the appendix lumen and/or wall can provide more information to increase the confidence level in diagnosing appendicitis. Our study's findings further demonstrate that in situations where AA is suspected, DW imaging is not always required.
